# Etymologia: Antimony

**DOI:** 10.3201/eid2408.ET2408

**Published:** 2018-08

**Authors:** Mark D. Walker

**Keywords:** etymologia, antimony, antimony potassium tartrate, schistosomiasis, leishmaniasis, trypanosomiasis, parasites, John Brian Christopherson

## Antimony [an′tĭ-mo′′ne]

One hundred years ago, John Brian Christopherson (1868–1955) discovered that antimony potassium tartrate (Figure) was an effective treatment against schistosomiasis. Antimony had been previously used against visceral leishmaniasis, *Trypanosoma brucei gambiense*, and yaws. The ancient Egyptians used antimony paste as mascara. In the Middle Ages, it was used as a laxative, which after swallowing and retrieval, could be reused. Alchemists used it to harden lead.

**Figure:**
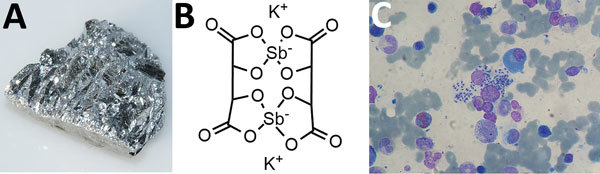
A) Antimony, unknown author, http://images-of-elements.com/, CC BY 3.0, https://commons.wikimedia.org/w/index.php?curid=9084452; B) Antimony potassium tartrate trihydrate, Chargelot, own work, CC BY-SA 4.0, https://commons.wikimedia.org/w/index.php?curid=47342907; C) Bone marrow aspiration: Leishmaniasis (Leishmania sp.) in liver transplant recipient, Paulo Henrique Orlandi Mourao, 2009, https://en.wikipedia.org/wiki/Leishmaniasis#/media/File:Leishmania_2009-04-14_smear.JPG;

Its name might have been derived from the Egyptian word for the metal *sdm*, from which the Greek *stimmi*, then the Latin *stibium*, then the French *antimoine* were derived. A more interesting, but unlikely, origin is that the French *antimoine* translates as monk’s killer, referring to its toxicity to religious alchemists. Antimony potassium tartrate remained the treatment of choice for schistosomiasis until the development of praziquantel in the 1980s.
